# The Nucleoid Binding Protein H-NS Biases Genome-Wide Transposon Insertion Landscapes

**DOI:** 10.1128/mBio.01351-16

**Published:** 2016-08-30

**Authors:** Satoshi Kimura, Troy P. Hubbard, Brigid M. Davis, Matthew K. Waldor

**Affiliations:** Division of Infectious Diseases, Brigham and Women’s Hospital, and Department of Microbiology and Immunobiology, Harvard Medical School and HHMI, Boston, Massachusetts, USA

## Abstract

Transposon insertion sequencing (TIS; also known as TnSeq) is a potent approach commonly used to comprehensively define the genetic loci that contribute to bacterial fitness in diverse environments. A key presumption underlying analyses of TIS datasets is that loci with a low frequency of transposon insertions contribute to fitness. However, it is not known whether factors such as nucleoid binding proteins can alter the frequency of transposon insertion and thus whether TIS output may systematically reflect factors that are independent of the role of the loci in fitness. Here, we investigated whether the histone-like nucleoid structuring (H-NS) protein, which preferentially associates with AT-rich sequences, modulates the frequency of Mariner transposon insertion in the *Vibrio cholerae* genome, using comparative analysis of TIS results from wild-type (wt) and *Δhns V. cholerae* strains. These analyses were overlaid on gene classification based on GC content as well as on extant genome-wide identification of H-NS binding loci. Our analyses revealed a significant dearth of insertions within AT-rich loci in wt *V. cholerae* that was not apparent in the *Δhns* insertion library. Additionally, we observed a striking correlation between genetic loci that are overrepresented in the *Δhns* insertion library relative to their insertion frequency in wt *V. cholerae* and loci previously found to physically interact with H-NS. Collectively, our findings reveal that factors other than genetic fitness can systematically modulate the frequency of transposon insertions in TIS studies and add a cautionary note to interpretation of TIS data, particularly for AT-rich sequences.

## IMPORTANCE

Transposon insertion sequencing (TIS) is often used to assess the relative frequency with which genetic loci can be disrupted, which is taken as an indicator of their importance for bacterial fitness. Here, we report that biological factors other than the relative levels of fitness of insertion mutants can influence TIS output. We found that the presence of the DNA binding protein H-NS, which preferentially recognizes AT-rich sequences, is linked to significant underrepresentation of mutations within AT-rich loci in transposon insertion libraries. Furthermore, there is a marked correspondence between loci bound by H-NS and loci with an increased frequency of disruption in a Δ*hns* insertion library relative to a wt library. Our data suggest that factors other than genetic fitness (e.g., DNA binding proteins such as H-NS) can systematically modulate the frequency of transposon insertions in TIS studies and add a note of caution for interpretation of TIS data.

## Observation

Transposon insertion sequencing (TIS) is a powerful tool purported to enable the unbiased and comprehensive identification of genetic loci required for bacterial fitness (1, 2). TIS employs deep sequencing of transposon insertion sites within a complex population of insertion mutants in order to determine the frequency with which genetic loci are disrupted. Subsequent genome-wide statistical analysis of relative insertion frequency enables identification of loci underrepresented for transposon insertion (3). Transposon insertion typically confers a loss-of-function phenotype; consequently, loci with a low frequency of transposon insertion are often presumed to contribute to bacterial fitness under the conditions assayed. TIS has enabled efficient identification of genes variously termed “essential,” “domain essential,” or “underrepresented” that are thought to promote bacterial growth in a variety of bacterial species (1, 2). Reliable gene classification has been bolstered by extensive optimization of TIS analysis methodologies, which have all but eliminated the technical artifacts that bias the detection of insertion frequency (3). However, biological factors that may alter the frequency of insertion, irrespective of the contributions of the loci to bacterial fitness, remain largely unexplored.

Many TIS studies have employed Mariner-based transposons, which insert exclusively at TA dinucleotides without additional sequence site restrictions (3, 4). For example, several such screens were performed to identify loci that promote *in vitro* growth of *Vibrio cholerae*, the cause of the diarrheal disease cholera, as well as to identify loci required by the pathogen for intestinal colonization of infant rabbits, a model host (5–8). Approximately 16% of the 3,751 nonredundant protein-coding *V. cholerae* genes were reported to be required for optimal *in vitro* growth (7). However, these genes included several well-characterized virulence loci that are dispensable for *in vitro* fitness, including the cholera toxin-encoding genes *ctxAB* and the *tcp* operon that enables production of a colonization-linked pilus. A similar phenomenon was observed in Mariner-based TIS analysis of *V. parahaemolyticus*, wherein components of a virulence-linked type III secretion system (T3SS2) dispensable for *in vitro* growth were underrepresented for transposon insertion (9). These findings suggest that the frequency of insertion at a locus may reflect factors in addition to the fitness of corresponding insertion mutants; however, the nature of such factors has not been defined. Notably, the underrepresented virulence-associated loci were acquired via horizontal gene transfer, and, like many horizontally acquired sequences, they have GC content markedly lower than that of the ancestral chromosomes into which they have integrated (10, 11).

In many bacterial species, horizontally acquired sequences are bound by the histone-like nucleoid structuring (H-NS) protein (12), which preferentially associates with AT-rich sequences through recognition of structural features of the minor groove of the DNA helix (13). H-NS can oligomerize along, or form cross-bridges between, AT-rich regions, thereby producing filamentous nucleoprotein complexes and stabilizing DNA hairpins (14). H-NS binding is associated with transcriptional silencing, due both to reduced access of transcription machinery to H-NS-bound promoters and to Rho-dependent transcriptional termination resulting from increased transcriptional pausing in bound regions (13). H-NS-mediated repression is overcome by the activity of transcription factors and other DNA binding proteins that compete with H-NS for target sites (12). For example, in *V. cholerae*, the virulence regulators ToxR and ToxT antagonize H-NS binding at virulence-related loci, including *tcpA* and *ctxA* (15–18). H-NS contributes to the regulation of diverse processes, and mutants lacking H-NS typically display attenuated growth, which in some cases has been specifically linked to overexpression of horizontally acquired sequences (19). H-NS’s recognition and silencing of horizontally acquired sequences are thought to offset the fitness cost of acquiring genes that are not governed by endogenous regulatory networks.

The known association between H-NS and many horizontally acquired sequences prompted us to consider the possibility that H-NS binding might account for the dearth of insertion mutants recovered for these loci. Previous studies have linked H-NS to transposition; for example, H-NS promotes the activity of Tn*10* through interactions with transpososome proteins (20). Additionally, H-NS is thought to modify IS*903* target site selection, although this conclusion is based on an analysis of relatively few insertion sites conducted prior to the availability of high-throughput sequencing (21). Despite these precedents and the fact that Mariner family transposons are widely used in TIS studies, the potential impacts of H-NS or similar nucleoid binding proteins on Mariner insertion have not been explored.

Here, we performed Mariner-based transposon mutagenesis of a *V. cholerae* H-NS mutant and implemented multiple statistical analyses in order to identify H-NS-dependent changes in transposon insertion profiles relative to a wild-type *V. cholerae* transposon library. We observed a striking correlation between genetic loci that display an H-NS-dependent reduction in transposon insertion and those previously found to interact with H-NS. H-NS-bound sequences with an H-NS-dependent low frequency of insertions are largely AT-rich loci, and they include numerous horizontally acquired elements and virulence-linked genes. Our findings suggest that H-NS binding biases the frequency of transposon insertion at certain loci and thus add a cautionary note to interpretation of TIS data.

**FIG 1:**
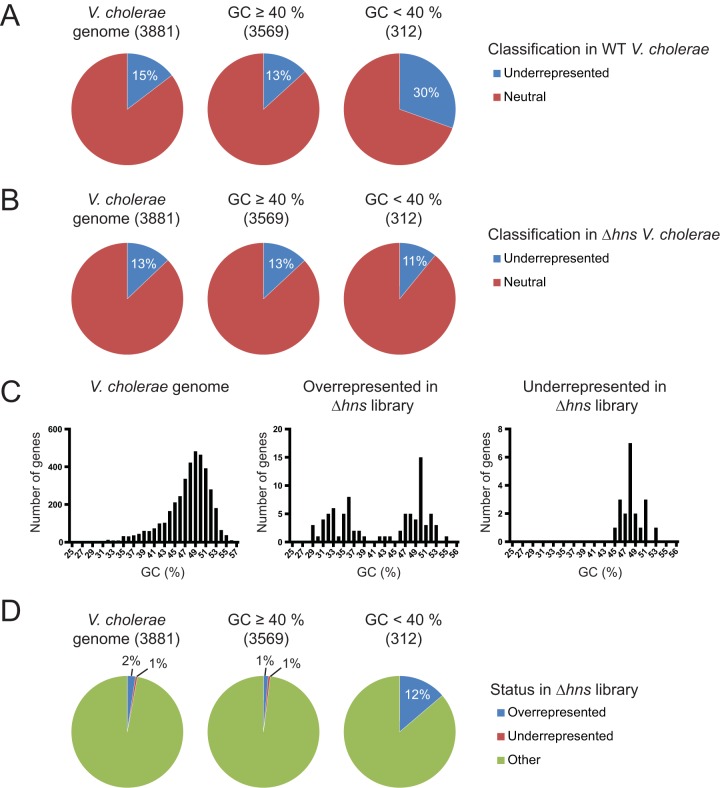
The skew in underrepresented transposon insertions in low-GC-content *V. cholerae* genes is not observed in the absence of H-NS. (A) Pie graphs depicting the relative frequencies of *V. cholerae* genes from reference 22, now classified as underrepresented or neutral. Frequencies are depicted for all classified genes and for the subsets of these genes with GC content of ≥40% and <40%. (B) Pie graphs depicting the relative frequencies of underrepresented and neutral loci among all genes categorized in the Δ*hns* library and for the subsets of these genes with GC content of ≥40% and <40%. (C) Histograms of GC content for all *V. cholerae* protein coding sequences, underrepresented genes in the Δ*hns* library, and overrepresented genes. (D) Pie graphs showing the relative frequencies of genes that are overrepresented or underrepresented in the Δ*hns* library relative to all *V. cholerae* open reading frames that could be classified and to the subsets of the genes with GC content of ≥40% and <40%.

A significant association between H-NS binding and low recovery of insertion mutants is likely to manifest as a relatively low frequency of insertion within AT-rich regions of the genome. To explore this possibility, we used the EL-ARTIST pipeline (5) to perform hidden Markov model (HMM)-based analyses of a *V. cholerae* TIS data set characterized in an earlier study (22) and classified genes based on transposon insertion frequency as well as GC content. HMM-based gene classification identified genetic loci underrepresented for transposon insertion. Notably, these genes, which correspond well to the “growth-promoting” loci identified in a similar analysis by Chao et al. in 2013 (7), are disproportionately AT rich (defined as GC content of <40%) (Fig. 1A; Fisher’s exact test, *P* = 3.9 × 10^−14^). Similarly, analysis of a *V. parahaemolyticus* insertion library (9) revealed that genes with a relatively low frequency of insertion are overrepresented among AT-rich loci (see Fig. S1 in the supplemental material; Fisher’s exact test, *P* = 3.6 × 10^−11^). Collectively, these associations between AT-rich DNA and a low frequency of transposon insertion are consistent with a possible linkage between H-NS binding and a bias in transposon insertion.

To investigate if H-NS contributes to the low frequency of insertion in AT-rich genetic loci, we performed TIS on a high-density transposon insertion library (70% of TA sites disrupted) generated in a *Δhns* derivative of *V. cholerae*. As described above, the EL-ARTIST pipeline (5) was used to analyze the sequence data and identify genetic loci underrepresented for transposon insertion (relative to the genome overall) in the *Δhns* library; gene classification as a function of AT content was also assessed. In striking contrast to the wild-type (wt) library, the *Δhns* library did not exhibit a correlation between low frequency of insertion and AT content (Fig. 1B; chi-square test, *P* = 0.30). In the *Δhns* library, genes underrepresented for transposon insertion were proportionately distributed between AT-rich genes and those with higher GC content. The absence of an AT content-correlated skew in the distribution of insertions in the *Δhns* library is consistent with the idea that H-NS accounts for the bias against insertion in low-GC-content genes observed in the distribution of transposon insertions in the wt strain.

**FIG 2:**
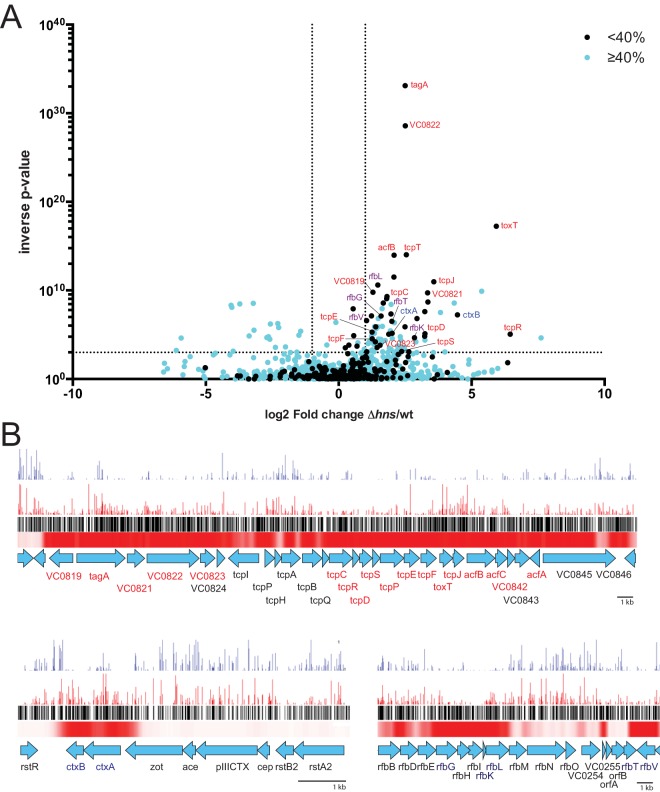
(A) Volcano plot of the results from Con-ARTIST analysis comparing insertions in the wt and Δ*hns* libraries. Genes with GC content of 40% or less are plotted in black; genes with GC content of ≥40% are plotted in cyan. Genes from the TCP-encoding VPI are labeled in red, cholera toxin-encoding genes are labeled in blue, and O-antigen biosynthetic genes are labeled in purple. (B) Artemis plots showing normalized frequencies of transposon insertion across the horizontally acquired regions encoding TCP, cholera toxin, and O-antigen. The insertion frequencies per locus are depicted with blue and red vertical lines for the wt and Δ*hns* libraries, respectively. Black lines depict all potential TA insertion sites. A heat map showing H-NS binding as determined by ChIP sequencing (23) is shown in red.

To compare the insertion profiles of the wt and *Δhns* libraries at the level of individual genes, we used the Con-ARTIST pipeline (5), which controls for stochastic factors in order to quantify differences between the transposon insertion profiles of two TIS data sets (Fig. 1D; see also Table S1 in the supplemental material). This analysis identified 84 genes that were overrepresented for transposon insertion in the Δ*hns* library (this study) relative to the wild-type C6706 library (22) (fold change, >2, *P* = <0.001; see Table S2) as well as 20 genes that were underrepresented among Δ*hns* insertion mutants (fold change, <0.5, *P* = <0.001; see Table S3). In contrast to the composition of the *V. cholerae* genome overall, in which <10% of genes are AT rich, overrepresented genes had a bimodal distribution of GC content, with 38 genes (45%) that were AT rich (Fig. 1C). These data reflect the fact that overrepresented genes in the Δ*hns* library are far more prevalent among AT-rich genes than in the remainder of the genome (12% versus 1%; Fig. 2A) (Fisher’s exact test, *P* = 5.4 × 10^−25^). Thus, direct comparison of the two transposon insertion libraries is consistent with the idea that the frequency of insertion mutations within AT-rich loci is modulated by the presence of H-NS. However, H-NS did not markedly affect mutation frequency within the portion of the genome with GC content of ≥40%.

To further explore the relationship between overrepresented genes and H-NS binding, we overlaid our TIS data on data from H-NS binding sites in the *V. cholerae* genome previously identified via chromatin immunoprecipitation (ChIP) sequencing (23). All 38 AT-rich overrepresented loci had detectable H-NS binding (see Table S2 in the supplemental material). In contrast, none of the 46 overrepresented genes with neutral GC content were bound by H-NS (see Table S2). H-NS binding was also not detected for the 20 underrepresented genes, all of which have GC content (44% to 53%) comparable to that of the *V. cholerae* genome (47.5%). We propose that H-NS-dependent changes in transposon insertion at the regions with neutral GC content reveal synthetic fitness phenotypes, consistent with the traditional interpretation of TIS data whereby differential insertion frequencies indicate altered fitness. Thus, not all H-NS-dependent changes in transposon insertion are the result of H-NS binding; however, the negative correlation between H-NS binding and transposon insertion (see Table S2) strongly suggests that interactions between H-NS and its targets can markedly reduce the frequency with which insertional disruption of these sites is observed.

The genes overrepresented in the Δ*hns* library and directly bound by HNS include a variety of horizontally acquired virulence loci. For example, they include *ctxA* and *ctxB*, which encode the A and B subunits of cholera toxin and are 2 of 3 genes in the horizontally acquired CTX prophage with GC content below 40% (38.5% and 32.5%, respectively) (Fig. 2B). Also, 17 of 24 AT-rich genes encoded within the horizontally acquired vibrio pathogenicity island (VPI) were overrepresented in the Δ*hns* library, including 9 associated with biosynthesis of toxin-coregulated pilus (TCP), a pilus critical for *V. cholerae* intestinal colonization (Fig. 2B). Previous studies have demonstrated that disruption of these loci, which are not expressed by *V. cholerae* under typical laboratory conditions at least in part due to H-NS-mediated repression (18), does not affect *in vitro* growth. Overrepresented loci also included components of the *rfb* operon, which enables O-antigen synthesis and was likely acquired through lateral gene transfer (Fig. 2B). As observed for the previously described virulence loci, *rfb* genes are not required for optimal growth of *V. cholerae* in vitro, but, unlike other virulence-linked loci, *rfb* genes display robust expression *in vitro* both in wt cells (where H-NS is bound) and in a *Δhns* mutant (24). Overall, these observations provide further support for the idea that H-NS can modulate the output of TIS-based studies and may particularly influence results for virulence genes, since these loci are often bound by H-NS.

There are several mechanisms by which H-NS may limit isolation of insertion mutants for the genetic loci to which it binds. H-NS could structurally occlude access of the Himar1 transpososome to its TA dinucleotide targets, either by polymerizing along the surface of AT-rich DNA and creating a protein barrier or by inducing formation of folded DNA structures that restrict transposition. There is precedence for the idea that other DNA binding proteins, such as ParB, can antagonize transposition of other transposons, such as Mu (25). Alternatively, H-NS might interfere with expression of the transposon-integral selectable marker when it has been inserted into transcriptionally repressed regions. The low frequency of insertion within the *rfb* operon, despite the constitutive and H-NS-independent expression of this region (24), suggests that transcriptional silencing is unlikely to account for the H-NS-dependent bias in insertion at this locus. It is unclear whether observations regarding the *rfb* operon are generalizable, particularly since H-NS is not typically bound to actively transcribed genes (19). Ultimately, it is difficult to exclude the possibility that elevated transcription of loci ordinarily silenced by H-NS contributes to the observed increases in insertions in such loci in the absence of H-NS. However, since there is not a positive correlation between gene expression levels and the frequency of transposition (see Fig. S2A in the supplemental material), relief of the silencing of H-NS bound genes is unlikely to account for the changes in Mariner insertion profiles that we observed in the absence of this nucleoid binding protein.

It is noteworthy that H-NS reportedly binds to many loci that do not display H-NS-dependent changes in transposon insertion. The 38 overrepresented AT-rich loci are only a small portion of the 332 loci bound by H-NS in *V. cholerae*; thus, H-NS binding does not inevitably impair recovery of associated insertion mutations. However, the genes overrepresented in the Δ*hns* strain generally had a higher degree of H-NS binding than genes that did not exhibit increased Mariner insertion (see Fig. S2B in the supplemental material). Besides the density of bound protein, the effects of H-NS on TIS output may be modulated by several factors, including the structure and stability of the DNA-H-NS complex, the prevalence of proteins that compete with H-NS for target sites, and/or the proximity of H-NS binding to Mariner target sites. Further studies are warranted to precisely define the molecular factors that govern the interplay between H-NS binding and Mariner transposon insertion.

Regardless of the mechanism(s) that modulates Mariner insertion into H-NS bound DNA, our findings reveal that H-NS binding can skew TIS-based assessment of AT-rich genes and, consequently, that caution in interpretation of TIS results for this subset of genes may be particularly warranted. It is possible that other nucleoid binding proteins, such as Fis, HU, or Rok, or potentially site-specific DNA binding proteins may also bias the distribution of transposon insertions and thus complicate interpretation of TIS data. While it might be possible to mitigate the bias in Mariner insertion caused by H-NS, e.g., by carrying out *in vitro* transposition in species that can be transformed at high frequency, it is unlikely that it will be possible to eliminate all biases in transposon insertion. Thus, elucidation of the biological processes that modify transposon site selection will enhance our capacity to interpret TIS experiments.

### Strains and medium.

All strains were grown on LB (1% NaCl). Antibiotic concentrations were 200 µg/ml streptomycin (Sm), 50 µg/ml kanamycin (Km), and 50 µg/ml carbenicillin (Cb). Wild-type *V. cholerae* C6706 and *Escherichia coli* SM10 (lambda pir) harboring pSC189 or pCVD442 were cultured in LB with Sm and LB with Cb, respectively.

### Construction of *V. cholerae hns* deletion mutant.

The Δ*hns* strain was constructed by homologous recombination, using a derivative of the negatively selectable suicide vector pCVD442 as previously described (22). The targeting vector contained 500 bp of DNAs flanking each side of *hns*, which were cloned into pCVD442’s SmaI site using isothermal assembly. Mutant selection was performed as previously described (5). The list of primer sequences is in Table S4 in the supplemental material.

### Transposon insertion sequencing.

A Himar1 transposon library in Δ*hns V. cholerae* was created and sequenced as previously described (22), except that conjugation was extended to 10 h. Sequenced reads were mapped onto a *V. cholerae* reference genome (N16961), and all TA sites were tallied and assigned to annotated genes as previously described (5). The library contained ~600,000 colonies with 134,064 unique transposon insertions, representing 70% of all TA dinucleotides from 5,300,801 mapped reads. The Con-ARTIST pipeline (5) was used to compare the Δ*hns* library with a previously characterized wild-type library (22) in order to identify overrepresented and underrepresented genes.

The raw data from ChIP sequencing (23) were plotted using Artemis (Fig. 2B).

### Accession number.

The raw sequencing reads of the three *hns* knockout libraries were deposited in Sequence Read Archive (SRA) in NCBI under accession numbers SRP081158, SRP081163, and SRP081165.

## SUPPLEMENTAL MATERIAL

Figure S1  Underrepresented transposon insertion in low-GC-content genes in *V. parahaemolyticus*. Download Figure S1, PDF file, 1.7 MB

Figure S2  (A) Genes underrepresented for transposon insertion in wt *V. cholerae* (data are from reference 22) are generally highly expressed (data are from reference 26). (B) Distribution of H-NS binding (by ChIP analysis; data are from reference 23) in genes overrepresented for insertion in the Δ*hns* strain (upper panel) and in the entire *V. cholerae* genome (lower panel); only the genes bound by H-NS are plotted in these graphs. Download Figure S2, PDF file, 1.7 MB

Table S1  Comparisons of transposon insertion data from Δ*hns* and wild-type (WT) *V. cholerae* (WT data are from reference 22). Con-ARTIST was used to compare the two data sets. El-ARTIST (5) was used to generate the hidden Markov model (HMM) categorizations. ChIP sequencing (ChIP-Seq) data (from reference 23) are also included. Table S1, XLS file, 1.2 MB

Table S2  List of genes overrepresented in Δ*hns* transposon insertion data set. Table S2, XLS file, 0.2 MB

Table S3  List of genes underrepresented in Δ*hns* transposon insertion data set. Table S3, XLS file, 0.2 MB

Table S4  List of primer sequences. Table S4, XLS file, 0.02 MB
