# Supplementary figures and images for: The Nucleoid Binding Protein H-NS Biases Genome-Wide Transposon Insertion Landscapes
Source: mBio. 2016 Aug 30;7(4):e01351-16. doi: 10.1128/mBio.01351-16 (PMC4999555; doi:10.1128/mBio.01351-16)

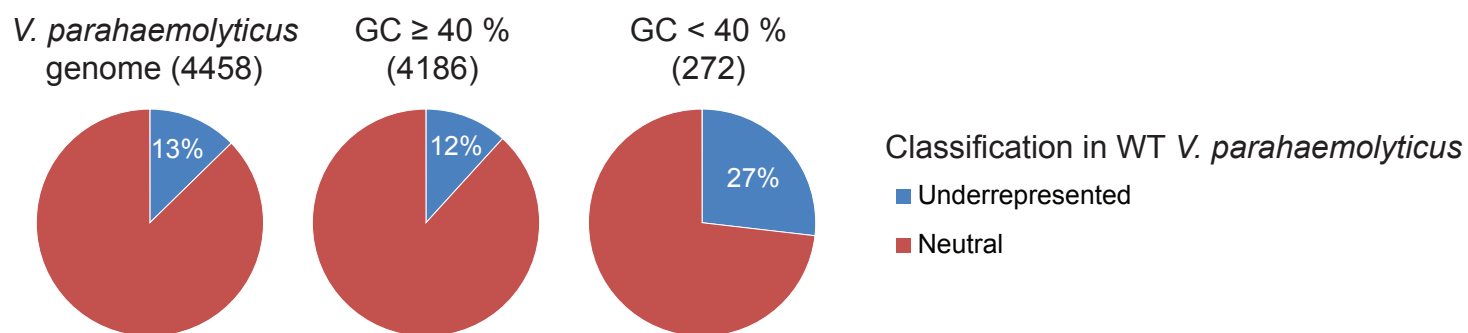

Supplementary Figure S1

Supplement: Figure S1 — Underrepresented transposon insertion in low-GC-content genes in V. parahaemolyticus. Download [file mbo004162965sf1.pdf]

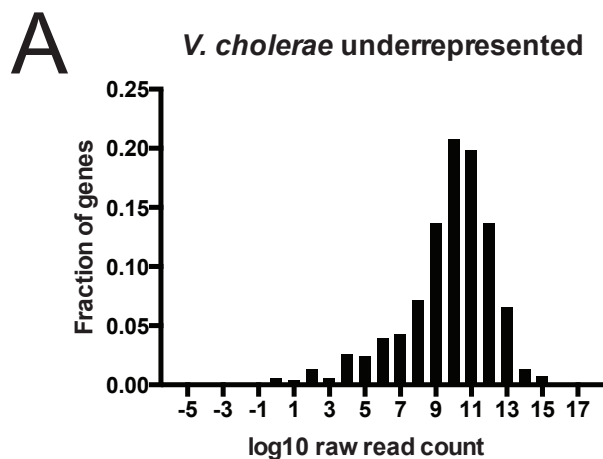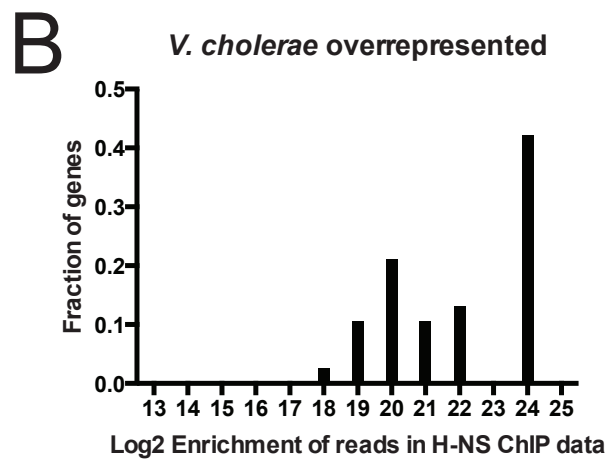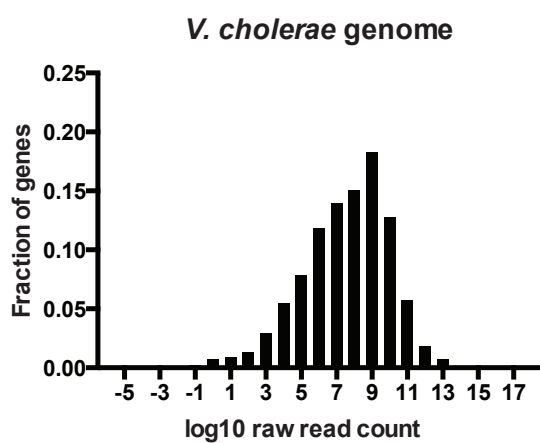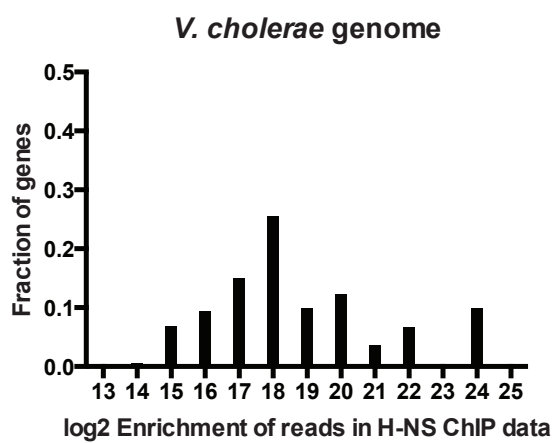

Supplementary Figure S2

Supplement: Figure S2 — (A) Genes underrepresented for transposon insertion in wt V. cholerae (data are from reference 22) are generally highly expressed (data are from reference 26). (B) Distribution of H-NS binding (by ChIP analysis; data are from reference 23) in genes overrepresented for insertion in the Δhns strain (upper panel) and in the entire V. cholerae genome (lower panel); only the genes bound by H-NS are plotted in these graphs. Download [file mbo004162965sf2.pdf]
